# Approaching Ventricular Tachycardia Ablation in 2024: An Update on Mapping and Ablation Strategies, Timing, and Future Directions

**DOI:** 10.3390/jcm13175017

**Published:** 2024-08-24

**Authors:** Andrea Di Cori, Lorenzo Pistelli, Matteo Parollo, Nicola Zaurino, Luca Segreti, Giulio Zucchelli

**Affiliations:** 1 Second Division of Cardiology, Cardiac-Thoracic and Vascular Department, University Hospital of Pisa, 56124 Pisa, Italy; 2 Biosense Webster, Johnson & Johnson Medial SpA, 00071 Pomezia, Italy

**Keywords:** ventricular tachycardia, ventricular tachycardia ablation, electro-anatomical mapping, computed tomography, cardiac magnetic resonance, artificial intelligence

## Abstract

This review provides insights into mapping and ablation strategies for VT, offering a comprehensive overview of contemporary approaches and future perspectives in the field. The strengths and limitations of classical mapping strategies, namely activation mapping, pace mapping, entrainment mapping, and substrate mapping, are deeply discussed. The increasing pivotal relevance of CMR and MDCT in substrate definition is highlighted, particularly in defining the border zone, tissue channels, and fat. The integration of CMR and MDCT images with EAM is explored, with a special focus on their role in enhancing effectiveness and procedure safety. The abstract concludes by illustrating the Pisa workflow for the VT ablation procedure.

## 1. History

Since A. Couch first reported the case of a recurrent ventricular tachycardia (VT) that was successfully terminated after the excision of a ventricular aneurysm, the idea of excluding ventricular arrhythmic substrates for terminating VT and preventing recurrences has taken hold [1]. In the following years, the effectiveness of surgical endocardial substrate excision for arrhythmogenic substrate removal in patients with a history of myocardial infarction was extensively investigated. However, studies have revealed the limited effectiveness of the surgical approach, which was burdened by a high rate of complications and mortality of up to 30% [2,3]. Concurrently, alongside endocardial excision procedures, the first studies on endocardial electrophysiological mapping during VT were conducted. These studies demonstrated the utility of electrophysiology (EP) studies in identifying the origin of VT, establishing an initial link between ECG morphology and the site of the VT origin [3,4,5]. Later, Geoffrey et al. reported three cases of VT that were effectively treated by delivering 300 J shocks through the tip of a 7 French quadripolar catheter, opening the era of VT endocardial ablation [5,6,7]. Subsequently, radiofrequency ablation emerged as a solution to address the issue of barotrauma associated with shock ablation [2,8]. The development of saline-irrigated catheters allowed for the creation of larger and deeper lesions and reduced the risk of clot formation, while the evolution of mapping tools and technologies contributed to improvements in the effectiveness and accuracy of VT catheter ablation in the following years [2,8].

## 2. Surface ECG and VT Origin

A twelve-lead surface ECG gives important insights about the origin of the tachycardia, guiding electrophysiological mapping. However, a surface ECG is influenced by many factors, such as heart rotation, the presence of structural abnormalities (e.g., fibrosis), the patient’s physical habitus, and lead placement [9]. Furthermore, in the context of reentrant arrhythmias, ECG accuracy is lower compared with focal arrhythmias, as the critical site does not always coincide with the exit site. Nevertheless, ECG recordings of VT still remain the source of relevant information for guiding ablation. The right or left bundle branch block aspect, axis of the QRS, transition in the precordial leads, and QRS width are key features for assessing the probable origin of the VT. While a right bundle branch block (RBBB) pattern is indicative of a left ventricle (LV) origin of the VT, a left bundle branch block (LBBB) aspect suggests a right ventricle or septal origin [9,10]. An inferior QRS axis is typically observed in VT originating from the upper quadrants; conversely, a superior axis suggests an inferior site of origin [9,10].

The earlier the positive to negative transition in the precordial leads, the more apical the origin. Typically, an RBBB pattern with a negative concordance in the precordial leads identifies an apical origin of the VT. The R/S amplitude ratio in V6 is helpful in distinguishing the possible origin, as a higher ratio suggests a more basal origin, while an R/S of less than 1 makes a middle ventricle origin more likely [9,10]. However, when an LBBB aspect with a transition later than V4 is present, a focus proximal to the right ventricle free wall should be suspected [9,10]. The QRS width also provides important information; when the source is near the conduction system (e.g., septal or near the fascicles), the QRS interval is narrow. In contrast, a free wall origin (or an epicardial origin) is suggested when the QRS interval is wider [10]. Items suggestive of an epicardial origin include the presence of a “pseudo-delta” wave lasting >34 ms, a QRS interval width >200 ms, an intrinsecoid deflection >85 ms, and an R-S complex duration >121 ms [10].

More in detail:

The *postero-medial papillary muscle* exit presents with a right bundle branch block (RBBB) pattern, a left or north-west axis, and a precordial transition usually between leads V3 and V5 [11].

The antero-lateral papillary muscle shows an RBBB morphology, an inferior axis, and transition between leads V3 and V5. There may also be inferior lead discordance, with DII being negative and DIII being positive [11].

For the *tricuspid valve*, the morphology is left bundle branch block (LBBB), with positivity in the aVL and DI. The transition in the precordial leads and the QRS width can help to distinguish between septal and lateral tricuspid valve origins. Specifically, a narrower QRS with a transition before V3 indicates a septal origin, while a later transition and a wider QRS suggest an origin from the free wall [11].

The infero-septal left ventricular wall presents an RBBB pattern with an R/S ratio in V1 greater than 1 and an rS complex in the inferior leads [11].

Unfortunately, a 12-lead ECG is very often not available, and the only information available may be the EGM recordings from devices.

## 3. Mapping

Over the years, as for many other arrhythmias, VT induction has been the cornerstone of ablation. Four mapping strategies (activation mapping, entrainment mapping, pace mapping, and substrate mapping) have been developed to address various issues and to enable the most precise localization of the source of arrhythmia and the underlying mechanisms. Particularly, VT mapping may be different in the case of focal activation/micro-reentry or in a macro reentrant circuit with a protected isthmus of slow conduction [12]. These mapping strategies are summarized in Table 1.

### 3.1. Activation Mapping

Activation mapping basically consists in defining a myocardial activation sequence during tachycardia [13]. In the case of a focal arrhythmia, activation mapping aims to identify the electrogram with the earliest onset with respect to the subsequent QRS complex [13]. Typically, the electrogram precedes the QRS complex by 30 to 50 ms, and a QS morphology is often observed in the unipolar signal at this site [13,14].

Activation mapping also provides valuable insights in the context of reentrant circuits, facilitating the identification of critical isthmuses [13,14]. In scar-related VTs, where the reentrant circuit is shown to be either structurally or functionally determinate, activation mapping represents an effective strategy as it can identify functional lines of block [15].

However, it should be acknowledged that the feasibility of a complete activation mapping process can be as low as 30% to 10% in some cases, because VT is frequently hemodynamically unstable and requires immediate termination [14].

### 3.2. Pace Mapping

The pace mapping technique relies on the concept that the QRS morphology of the paced beat at the VT exit site is similar to the QRS of the targeted VT [14]. The higher the correspondence between the paced QRS and the reference QRS, the closer the paced site is to the VT exit site. Thus, some authors have determined that in the context of idiopathic focal VT, pace mapping could represent a surrogate for activation mapping [16].

On the other hand, in the context of macro-reentrant VT, perfect QRS matching may be observed over multiple sites with varying stimuli to the QRS interval (S-QRS interval). The shortest S-QRS interval is indicative of the circuit’s exit site, and the S-QRS interval becomes progressively longer as the paced site becomes closer to the isthmus. An abrupt change in the paced QRS morphology identifies the site at which transition across the slow-conduction zone occurs, identifying the mid-isthmus zone as proposed by De Chillou et al. [16]. This is a zone of extremely slow conduction that effectively divides the circuit into entrance and exit halves. 

While pace mapping is a valuable technique, especially in the case of focal VT and even in hemodynamically unstable VT, it does have the following limitations that should be considered:

(1) High correspondence (matching) between the paced and clinical QRS is required [14].
(2) Pace mapping involves qualitative analysis and may have poor reproducibility, particularly due to the lack of a universally accepted definition of a good correlation [12,16].
(3) Intramural circuits can limit the ability of pace mapping to identify critical isthmuses, as part of the circuit is not mappable [12].
(4) An abrupt change in the QRS morphology may not necessarily pinpoint the isthmus but could represent a remote bystander [12].
(5) Attention should be given to the paced rate and pacing amplitude, as the morphology of the paced QRS can also be influenced by the paced rate, and high pacing amplitudes may result in far-field captures [12].
(6) Stimulation is usually bipolar and, as such, it is less accurate in identifying the critical isthmus.

### 3.3. Entrainment Mapping

In the context of hemodynamically stable reentrant VT, entrainment mapping has proven to be effective in identifying the target isthmus. The isthmus is defined as the location where paced beats result in a concealed fusion, with a post-pacing interval (PPI) minus tachycardia cycle length (TCL) of less than 30 ms and an S-QRS interval that is less than 50% of the TCL. Some groups have reported success rates of up to 70% when RF ablation is performed at such a site [14,17].

However, in cases involving structural heart disease with fibrosis and reentrant circuits, bystander sites are frequently present. Bystander sites are located adjacent to the reentrant circuit but do not play an essential role in sustaining the arrhythmia. Due to their proximity to the isthmus, bystanders may mimic the isthmus during pacing maneuvers [14]. Differentiating sites directly involved in sustaining the arrhythmia from bystander areas is crucial for the procedure’s effectiveness. When the bystander site is relatively distant from the circuit, pacing at this site results in a distinct QRS morphology or a return cycle longer than the TCL [17]. However, when concealed pacing occurs with a PPI-TCL of less than 30 ms, pacing at progressively higher rates can help to distinguish the isthmus from the bystander site. In the latter case, dissociation of the electrogram from the QRS may be recorded [17].

Another potential source of error is the antidromic activation of the circuit when pacing at a site proximal to the isthmus. This can result in a completely different QRS morphology and a significantly different S-QRS interval, leading to measurement errors [17].

Similarly to activation mapping, entrainment mapping also requires a stable VT to be conducted effectively.

### 3.4. Substrate Mapping

Since up to 90% of VTs are hemodynamically not tolerated, activation and entrainment mapping may not always be feasible [18]. In such settings, substrate mapping becomes a necessary approach. Substrate mapping consists in searching for abnormal or low-voltage electrograms (namely late, split, or fragmented potentials, high-frequency potentials suggestive of local abnormal ventricular activity (LAVA), and low-voltage potentials) during sinus rhythm, as these electrograms reflect abnormal conduction sites [13]. Studies have demonstrated that abnormal ventricular activity during sinus rhythm is present at the site of VT origin in approximately 85% of cases [18]. *Low-voltage and fragmented electrograms* are often indicative of regions where electrical activity is dispersed; these are frequently localized within the scar border zone, creating conditions that favor the development of reentry mechanisms [19,20]. According to Anter et al., *LAVA* has limited accuracy (sensitivity and specificity of approximately 38% and 32%, respectively) [21]. However, data from Jais et al. reported effective LAVA RF ablation in terms of VT-free survival [22].

The advent of high-density mapping tools has undeniably improved substrate definition and enhanced the precise identification of LAVA, consistent with the lower recurrence rates of ventricular tachycardia (VT) observed with multipolar catheter usage [23]. High-density mapping facilitates more accurate identification and extensive elimination of LAVA, which correlates with reduced recurrence rates of VT [23]. *Late potentials (LPs)* are detected in electrograms as following the preceding QRS by 30 to 50 ms and are considered to indicate a viable ablation target, as they are thought to be indicative of a critical isthmus [20]. During sinus rhythm, a late potential represents a site of late activation that is distal to a zone of slow conduction during orthodromic activation of the circuit. However, LPs are not suitable targets in cases of functional block and reentry, as discussed below. Furthermore, LPs cannot distinguish whether the later activation recorded is part of the circuit or a bystander site located after the slow-conduction isthmus. Nevertheless, a single-center study from Vergara et al. described satisfying results from substrate VT ablation that targeted LPs [24,25]. These data have been recently confirmed by a prospective multicenter study [20].

Activation mapping of *isochronal late potentials (ILAM)* during sinus rhythm enables the identification of zones with contemporary late activation (isochrones). Interestingly, the zone with the latest isochrone (i.e., the zone with the latest LP) is rarely a site of effective ablation [26]. Instead, effective ablation is often found in correspondence with the penultimate and antepenultimate LP isochrone [26]. This is likely due to the fact that the latest LPs frequently represent signals from bystander sites, located far from the circuit and distal to the slow-conduction zone, which, in contrast, is an appropriate ablation target. Notably, based on their experience in high-resolution mapping, Josephson et al. identified the most pronounced conduction delay at the acute angle near the isthmus entrance, while the isthmus itself appeared to exhibit normal or slightly slowed conduction [15,20].

During ILAM, the slow-conduction zone is characterized by a high density of different isochrones, the so-called “isochronal crowding”. Isochronal crowding is defined by the presence of more than two isochrones in <1 cm [26]. Ablation at this site has shown effectiveness, with a recurrence-free survival of up to 73% at 22 months [27].

Importantly, abnormal electrograms are not consistently present in all patients with structural VT. Additionally, attempting to map the entire ventricle to identify these abnormal electrograms is impractical, primarily due to the time required. Therefore, voltage mapping, a key component of substrate mapping, plays a pivotal role in identifying areas more prone to underly arrhythmic genesis. It involves the use of both unipolar and bipolar mapping systems to distinguish between scar tissue and healthy tissue based on voltage amplitudes. The majority of ablation target sites have been identified within an area of dense scar or in the scar border zone [28,29]. A voltage amplitude higher than 1.5 mV is generally considered indicative of healthy tissue, while a voltage lower than 1.5 mV suggests the infarct border zone (from >0.5 mV to 1.5 mV) or scar tissue when lower than 0.5 mV [13,20]. Very low voltages (<0.1 mV) have been described as consistent with electrical inexcitability, while voltages between 0.1 mV and 0.5 mV, commonly described as dense scar, may still have some electrical activity, detectable only through high-resolution mapping using small electrodes with a low interelectrode distance [20].

Voltage mapping can also be influenced by the angle between the tissue and the catheter (“angle of incidence”) and the contact of the catheter tip with the tissue, potentially leading to false low voltage when unstable contact is present, as described by Blauer et al. [20,30]. Furthermore, conduction velocities between the two poles and the activation front are partially responsible for any false low voltages recorded [31]. However, limitations of bipolar voltage mapping alone do not fully account for the high recurrence rate, particularly in post-infarct VT, which can be as high as 50% at one year and 19% at 30 days [31,32].

Of note, post-infarct remodeling not only consists in fibrous tissue deposition but also myocyte disarray, modifications in gap junctions, and ion channel dysfunction. These factors collectively contribute to arrhythmogenesis by altering the anisotropic properties of the tissue, facilitating the development of functional blocks and functionally determined reentry circuits [20]. In the setting of functionally determined conduction blocks or slow conduction, substrate mapping does not provide enlightenment about any abnormal electrical activity within the reentrant circuit [20]. 

It is more likely that the limited effectiveness of substrate mapping is due to this aspect, especially in post-infarct VT, where the development of zones of functional block is common [21]. 

In this context, identifying areas of hidden slow conduction (HSC) or decremental conduction properties offers the opportunity to locate entrance channels and VT critical sites, thereby revealing the functional substrate underlying the normal bipolar voltage map [33,34]. Since the work of Lammers et al., decremental conduction and unidirectional conduction blocks have been shown to be linked properties, explaining why decremental properties are typical of critical VT sites [35]. 

Stressing conduction properties with a double extra-stimulus may reveal the presence of decremental conduction properties in areas where abnormal signals or late potentials may be hidden by ventricular far-field signals or where the ventricular electrogram appears normal due to the presence of a functional substrate [34]. By stressing the conduction in these zones, activation is delayed, revealing a late potential. A double extra-stimulus delivered during sinus rhythm is commonly used to reveal areas of HSC. Similarly, delivering a decremental extra-stimulus (usually set at VERP + 20 ms) during programmed ventricular stimulation may evoke areas of delayed activation that are not appreciable in sinus rhythm [33,36]. Activation mapping of these evoked delayed activations can identify sites critical for VT maintenance [33,36]. This technique is called decrement-evoked potential (DeEP) mapping. DeEP mapping has shown higher accuracy than identifying late potentials and, like HSC identification, it allows for a better definition of the functional substrate [34,36]. While late potentials have been shown to be of poor specificity, recent studies targeting HSC or delayed evoked activation at DeEP mapping for scar de-channeling have shown promising and interesting results [34,36].

## 4. Imaging

### 4.1. Cardiac Magnetic Resonance

As previously discussed, substrate mapping is susceptible for far-field artifacts, catheter-related issues, and catheter–tissue contact and angulation issues, which may result in inaccurate tissue voltage definitions [37]. Furthermore, it exhibits an extremely limited ability in defining deeper electrical activity, such as the presence of altered voltage amplitude in the midwall myocardium [37]. This limitation becomes even more relevant in the context of non-ischemic structural heart diseases, where arrhythmogenic sources are often located in the midwall, especially when involving the interventricular basal septum, a region particularly challenging to characterize by bipolar mapping [37].

Interestingly, areas of transmural LGE at CMR have been found to correlate with zones of low voltage amplitude (less than 1.5 mV) during voltage mapping, once again confirming the association between scar tissue and low voltages [37,38,39,40]. In more detail, regions exhibiting transmural scar and a transition zone (border zone) have been shown to host concealed entrainment sites and the majority of VT termination sites [41].

In a study by Wijnmaalen et al., it was noteworthy that all VT isthmuses were localized within scar tissue defined by LGE on CMR [40]. However, it is important to highlight that not all isthmuses were identifiable using bipolar mapping [40]. Similarly, Acosta demonstrated that in one out of three cases, slow-conduction zones may be concealed within normal voltages in voltage mapping, whereas these slow-conduction zones are frequently found in border zone tissue [34]. These findings further enlighten the significant role of CMR imaging in planning and executing successful VT ablation procedures. Finally, the time required to create an accurate electro-anatomical map should not be underestimated, as it demands both time and operator skills. Therefore, in recent years, the role of CMR has gained increasing relevance in the planning and execution of VT ablation procedures. Pre-procedural imaging allows for optimal approach planning and limits the electro-anatomical mapping (EAM) to previously identified areas of interest, thereby enhancing the procedural efficiency. This approach also assists in identifying those patients who would benefit from an epicardial approach, as the presence of subepicardial delayed enhancement has demonstrated high specificity in predicting an epicardial origin of VT [37].

As previously mentioned, VT ablation targets are frequently localized within scar and scar border zone areas.

Several studies have demonstrated CMR’s ability to precisely define the border zone and heterogeneous tissue channels by distinguishing between different LGE signal intensities [42]. The border zone may represent the transitional tissue between dense scar and the normal myocardium or even exist within dense scar tissue, creating corridors that bridge the normal myocardium through scar tissue [42]. Such corridors have been found to correspond with voltage channels and late potentials identified by electro-anatomical mapping, and they account for the genesis of reentrant arrhythmias [37,42].

Based on this evidence, the 2019 European Heart Rhythm Association expert consensus statement on the catheter ablation of ventricular arrhythmias recommends performing pre-procedural planning with CMR to reduce recurrences (Class II, level of evidence A) [43]. 

In recent years, with technological advancements, the possibility of merging CMR images with EAM has transformed the ablation of VT, making the role of CMR significant not only in pre-operative planning but also during the procedure itself. Recent studies suggest that a substantial improvement in terms of freedom from VT recurrences can be achieved by integrating standard mapping strategies with real-time CMR [23,44]. 

Soto-Iglesias et al. took the next step, reporting for the first time the feasibility and effectiveness of CMR-guided VT ablation in 28 patients with scar-related VTs [45]. CMR-guided VT ablation was compared with CMR-aided and non-CMR VT ablation, demonstrating significantly shorter procedural and fluoroscopy times compared with both CMR-aided (*p* < 0.001 for both comparisons) and non-CMR procedures (*p* < 0.001 for both comparisons) [45]. Furthermore, it exhibited superiority in terms of VT inducibility (*p* = 0.04) and the one-year recurrence rate (*p* = 0.019) when compared with non-CMR VT ablation [45]. Although no statistically significant differences were found between CMR-aided and CMR-guided ablation, the latter exhibited slightly lower VT inducibility (18% vs. 32%) without significant differences in terms of procedure safety [45].

**Figure 1:**
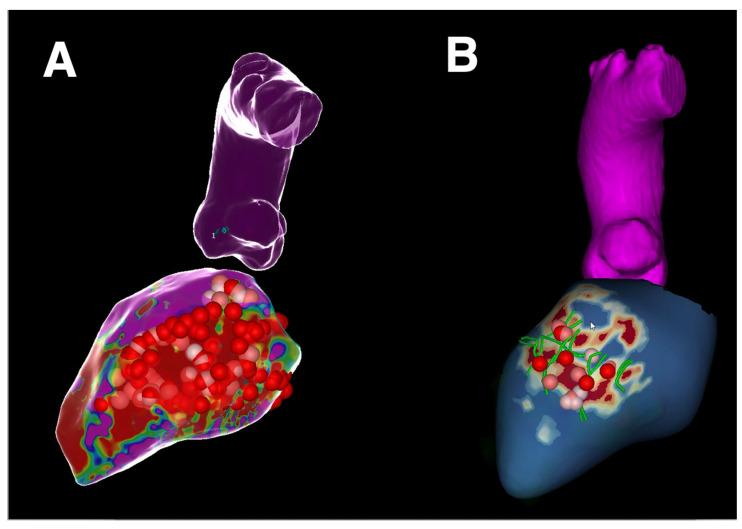
Different approaches for VT ablation. (**A**) Bipolar voltage map of the left ventricle showing ablation tags according to the scar homogenization technique. (**B**) CMR-derived substrate map of the left ventricle obtained with ADAS3D processing (blue: healthy tissue; pink: border zone,; red: dense scar) showing heterogeneous tissue channels (in green) with ablation tags according to the CMR-guided scar dechannelling technique. VT: ventricular tachycardia; CMR: cardiac magnetic resonance.

Of course, larger studies and, ideally, randomized clinical trials are needed to accurately assess whether the CMR-guided strategy is superior to more conventional approaches. An ongoing multicenter clinical trial is currently investigating CMR-aided and CMR-guided approaches in comparison with traditional substrate-driven ablation strategies [46] (Figure 1).

Since most patients undergoing VT ablation are ICD carriers, this approach may be limited by cardiac device-related artifacts in CMR, which can lead to inadequate image quality and compromise accuracy in the LGE assessment [47]. However, the use of wideband LGE sequence acquisition has proven to be effective in removing artifacts in up to 87% of cases [48,49].

### 4.2. Multi-Detector Computed Tomography

While CMR provides extremely accurate tissue characterization and can identify scars and corridors, multi-detector computed tomography (MDCT) enables precise anatomical definition with significantly higher spatial resolution [50]. This is particularly important in the context of epicardial ablation, as it allows for the definition of coronary artery courses and the location of the left phrenic nerve, enhancing procedural safety [50,51]. Additionally, MDCT accurately detects calcium deposits and defines the distribution of epicardial fat [50]. This aspect is crucial in procedural planning, as fat deposits may lead to false voltage attenuation and ablation failure, essentially acting as a shield that protects the target site from RFs. Despite MDCT’s limits in scar characterization compared with cardiac magnetic resonance, it is still as a valuable alternative, especially in cases where CE MRI is contraindicated, suboptimal, or unavailable [23,37]. Despite its inability to allow direct assessment of myocardial fibrotic substrates, MDCT’s high spatial resolution enables precise LV wall thickness analysis. In a paper by Komatsu et al., regions with a wall thickness of less than 5 mm had high correlation with endocardial low-voltage areas in ischemic subjects. Wall thickness analysis is, however, burdened by a strong relationship with the transmurality of myocardial damage. In subjects with predominantly subendocardial or subepicardial myocardial remodeling, the wall thickness variations are subtle, and CT channels are less common [52].

Lipomatous metaplasia (LM), found both in ischemic and non-ischemic heart disease, is another specific histological feature of the jeopardized myocardium, and its contribution to arrhythmogenesis has recently been scrutinized. MDCT can reliably identify LM with high spatial resolution, which has potential implications for mapping and ablation guidance [53].

The utilization of late-iodine-enhanced MDCT for delineating fibrotic scar areas that correlate with EAM and successful ablation sites has been explored, and it is currently under investigation [54]. A proprietary software known as the Multi-modality Platform for Specific Imaging in Cardiology (MUSIC) has been studied for its potential in guiding scar-related ventricular tachycardia (VT) ablation. An international consortium of institutes employing MUSIC/inHEART technology for VT ablation is actively assessing its impact on acute and long-term procedural outcomes involving RF and other treatment modalities [55].

## 5. Endocardial and Epicardial Approaches

Reentrant circuits responsible for ventricular arrhythmias are rarely confined to the endocardial layer. The large majority of reentrant ventricular arrhythmias involve the epicardium, showing a three-dimensional propagation of the arrhythmia wavefront through different myocardial layers [56]. The role of the epicardium in VT is of increasing interest.

According to data from Fernandez-Armenta et al., 63% of patients with infarct-related transmural scar have epicardial corridors, further corroborating findings from Di Biase and Tung [57,58,59].

These authors suggested a higher effectiveness of a combined endo-epicardial VT ablation procedure compared with the solely endocardial approach [58,59]. Noteworthily, in their studies, only half of the patients undergoing epicardial substrate mapping subsequently underwent epicardial ablation [58,59]. 

In contrast, Acosta selected patients for epicardial mapping only if they had clear evidence of transmural scar, resulting in a higher percentage of patients undergoing epicardial ablation following epicardial mapping (87.5%) [60].

These data reaffirm the strong connection between transmural scar and the epicardial arrhythmic substrate and highlight, once again, the utility of CMR in pre-procedural planning [57].

As previously mentioned, most reentrant circuits involve the epicardium.

**Figure 2:**
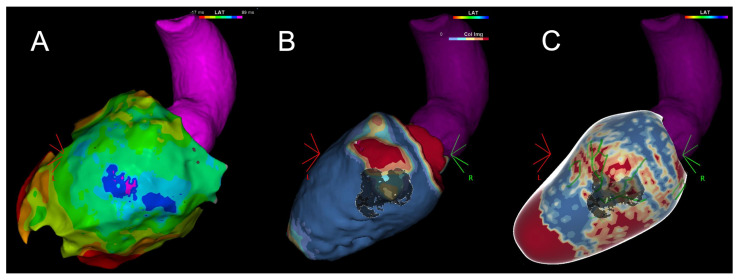
(**A**) Epicardial isochronal LAT map showing delayed potentials at the mid-basal inferior/inferolateral wall. (**B**) LVWT MDCT showing thinned areas and the superimposed lipomatous metaplasia. (**C**) LGE-CMR processed with ADAS3D showing the epicardial scar and border zone areas with heterogeneous tissue channels (green). LAT: local activation mapping, LVWT: left ventricle wall thickness, MDCT: multi-detector computed tomography, LGE-CMR: late gadolinium enhancement cardiac magnetic resonance.

In particular, up to 80% of infarct-related VTs and up to 77% of non-ischemic cardiomyopathy-related VTs are accounted for by reentrant circuits involving the epicardium (Figure 2) [58].

Historically, the boundaries of reentrant VT circuits have been conceived as a two-dimensional (2D) framework, and the distinction between fixed and functional boundaries has remained unresolved. In most VT circuits, fixed lines of block (LOBs) can be identified through intrinsic or paced activation during sinus rhythm. An innovative approach involves analyzing activation patterns while pacing within the scar substrate, which may reveal concealed boundaries previously thought to be of a functional nature. When viewed from the perspective of the myocardial surface, these LOBs are often associated with intramural conduction, supporting the notion of a three-dimensional (3D) hyperboloid structure for VT circuits [61]. 

A better understanding of specific arrhythmic substrates under different etiologies is crucial in developing an ablation strategy tailored to the specific underlying disease.

Subxiphoid “dry” epicardial access to the virtual pericardial space can be challenging and has been associated with an overall complication rate that ranges from 6% to 25%. Among these complications, inadvertent RV puncture is the most common. In a recent multicenter registry, intentional coronary vein exit with CO_2_ insufflation into the epicardial space was shown to be an effective strategy for enhancing the safety of subxiphoid epicardial access, virtually eliminating the risk of RV puncture. Such promising feasibility and safety data were confirmed in subsequent single-center experiences [62]. Through this strategy, cases of significant bleeding are infrequent, especially if the exit site is created in the distal part of the vein and before heparin administration [63]. 

## 6. Deferred vs. Early Ablation

The VANISH trial demonstrated the superiority of RF ablation over escalating antiarrhythmic drugs in terms of survival or survival free from arrhythmic storms or ICD shocks at 30 days in patients with ischemic cardiopathy and VTs [64]. This raised the question about the optimal timing for performing catheter ablation for structural VT.

Several randomized clinical trials have attempted to address this question in the past years (Table 2).

In the SMASH-VT trial, 128 patients with a history of myocardial infarction and a first episode of VT or ventricular fibrillation were randomly assigned to receive either ICD and VT ablation or ICD alone. At 23 months, 33% of the patients who did not undergo catheter ablation and 12% of the patients treated with catheter ablation experienced appropriated ICD shock (hazard ratio in the ablation group, 0.35; 95% confidence interval, 0.15 to 0.78; *p* = 0.007) [68]. The BERLIN VT trial, which enrolled patients with previous myocardial infarction and an ejection fraction between 30 and 50%, confirmed a lower burden of ICD shock among patients who underwent early catheter ablation (i.e., after the first episode of VT) (*p* = 0.020) [66]. However, it is worth noting that ICD shocks were a secondary outcome, and the study failed to demonstrate the superiority of early VT ablation under the primary outcome, which was a composite of all-cause deaths and unplanned hospitalizations [66]. Similar results to the BERLIN VT were obtained from the PAUSE-SCD trial among patients with cardiomyopathy and secondary prevention ICD implant indication [70]. 

A subsequent metanalysis showed how early catheter ablation for VT in patients with a history of myocardial infarction and an EF > 30% was effective in reducing ICD therapies, but it did not identify any benefit in terms of mortality [71]. However, a recent trial enrolling patients either with ischemic or dilated cardiomyopathy, the PARTITA trial, showed promising results for an early ablation approach in terms of mortality and hospitalization [65]. Nevertheless, recent trials such as PREVENT and PREVENTIVE VT are evaluating the role of prophylactic VT substrate ablation as the primary prevention in chronic post-MI patients with potentially arrhythmogenic scars [72,73].

In light of these studies, it seems reasonable to consider early ablation for patients with VT and structural heart disease in order to reduce recurrences, improve their quality of life, and potentially enhance their survival. Of note, unfortunately, in clinical practice, patients are frequently referred late, e.g., after the second or third episode of VT.

However, in some settings, such as in arrhythmogenic ventricular dysplasia, the evolving nature of the substrate makes recurrences very likely. In such cases, ablation should probably be considered a last resort for controlling VTs.

## 7. Procedural VT Ablation Workflow

Conventional VT mapping is effective at identifying critical sites responsible for inducible and stable VTs, but it may only capture a portion of the possible VTs related to scar tissue. In contrast, substrate mapping has the potential to identify any critical VT sites that result from conduction abnormalities or local electrogram irregularities. The reduced mortality and rehospitalization rates observed in the group undergoing substrate ablation may be due to fewer disruptions in their hemodynamics and fewer instances of VT recurrence [74]. Additionally, substrate ablation for scar-related VT has shown promising long-term clinical outcomes, with a prospective multicenter registry of 412 patients demonstrating a 1-year VT-free survival rate of 82.5% [75]. 

Regarding safety, a substrate-based approach is likely to reduce the need for electrical cardioversion of unstable VTs, decrease procedural time, and minimize radiation exposure for both patients and physicians. Two meta-analyses have also compared conventional VT ablation with substrate-based VT ablation in terms of long-term clinical outcomes [76,77] Although these analyses showed trends of lower VT recurrence and all-cause mortality with substrate ablation at 18–24 months of follow-up, they acknowledged the potential bias in randomization in many studies. Consequently, it is reasonable to conclude that, considering the higher baseline risk in the VT substrate group, substrate ablation appears to be a more favorable approach. Ongoing randomized studies are evaluating the impact of substrate imaging with pre-procedural imaging support.

The workflow for VT ablation should take into account factors such as the patient’s baseline risk (including comorbidities and heart disease), the procedural risk (whether it involves endocardial and/or epicardial approaches), and the procedural goal (such as clinical VT ablation or the elimination of ICD shocks or any inducible arrhythmia). 

**Figure 3:**
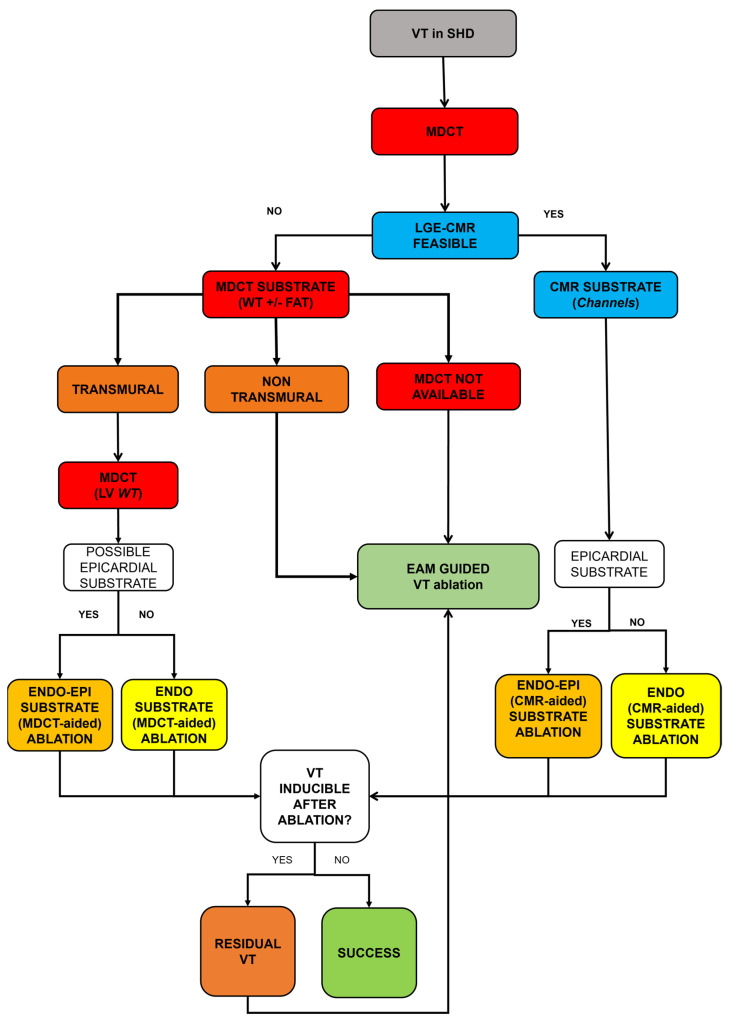
Pisa workflow for structural and nonstructural VT ablation. VT: Ventricular tachycardia, SHD: Structural heart disease, MDCT: Multi-detector computed tomography, LV: Left ventricle, WT: wall thickness, LGE-CMR: late gadolinium enhancement cardiac magnetic resonance, ENDO: Endocardium, EPI: Epicardium.

Figure 3 shows the Pisa workflow for structural and nonstructural VT ablation.

## 8. Complication

The VT ablation procedure carries a significant complication rate, ranging from 5% to 9%. This is partly due to patient characteristics and partly related to the procedure itself [78,79]. Older age, ischemic etiology, and heart failure are predictors of worse outcomes at the patient level [80]. Acute death (within the first few days after the procedure) occurs in 0.9% of cases, primarily due to VT recurrences (in up to one-third of cases) [78,79]. A European registry reported that up to 40% of patients who died after the indexed procedure had been admitted for an arrhythmic storm, and up to 25% still had inducible VT at the end of the procedure [78].

Vascular access complications account for approximately 10% of all major complications but have been reduced by up to 65% with the use of echographic guidance [78,79,81]. Hemodynamically relevant pericardial effusion has been observed in around 2.2% of patients undergoing VT ablation [78]. In nearly all these cases, drainage of the effusion was required, generally at the time of the indexed procedure [78]. Stroke is rare (0.4%) and is associated with the interruption of oral anticoagulation in 75% of cases [78].

## 9. Future Perspectives in VT Ablation

The primary objective of VT ablation is to enhance procedural efficacy while minimizing associated risks, and this relies on the ongoing development of mapping and ablation strategies.

Regarding mapping, the robust identification of optimal VT ablation targets currently involves invasive EP procedures, which introduce clinical risks, even with advanced techniques such as 3D EAM and other risk-mitigating approaches. Despite the capabilities of these strategies to accurately characterize clinical VT, their effectiveness may be constrained by challenges like non-inducible or non-sustained and hemodynamically poorly tolerated VTs. Consequently, there is a critical need for efficient non-invasive methods to localize VT circuits.

The surface 12-lead electrocardiogram (ECG) remains pivotal in guiding pre-procedure planning for VT ablation. Non-invasive approaches utilizing the ECG, such as ECG imaging (ECGi) and automated algorithms, are increasingly under investigation for use in VT localization. Additionally, electrogram (EGM) recordings stored in implantable electronic devices have proven valuable for guiding clinical and in silico pace mapping, aiding in the characterization of clinical VT and facilitating the automatic localization of focal VTs. Since most candidates for CA already have an implanted device, automating the post-infarct VT localization directly from EGM recordings could enhance the safety, speed, and success of CA. However, limitations arise from the failure to record an ECG during the clinical arrhythmia, and concerns exist regarding its precision and accuracy compared with invasive modalities.

A proposed computational deep learning (DL) framework could be employed for the direct localization of post-infarct VT exit sites from ECGs and/or implanted device EGMs, either in the absence of patient imaging data or in combination with computationally efficient, patient-specific modeling. This approach holds the potential to improve the safety and speed of pre-procedure ablation planning. Data collection from registries and large databases could contribute to the creation of a learning machine for VT circuit modeling and predictions [82]. 

Regarding ablation technologies, the field of VT ablation has seen significant advancements in mapping tools and catheters, leading to safer and more effective procedures. The use of decreased osmolarity irrigants and the possibility of delivering high-power energy allow for larger and deeper lesions [83]. Recently, a new catheter with an extendable–retractable 27-gauge needle at the tip proved to be helpful in mapping and ablating the intramyocardial substrate at the cost of an acceptable low risk of procedure-related complications [84]. 

While RF ablation remains the primary approach for VT ablation, alternative methods have been developed, particularly for challenging cases. 

In the context of an intramyocardial substrate or a summit origin of the VT, 98% ethanol injection through cardiac veins or coronary arteries may represent an option as a bailout procedure. This technique has shown relative safety and excellent outcomes [85,86,87].

Electroporation is an emerging ablation approach for atrial fibrillation. It causes irreversible damage to the cell membrane without incurring the risk of barotrauma.

Animal models have shown that epicardial electroporation ablation may allow for the creation of transmural lesions in the ventricular wall [88]. The main strength of electroporation is its high tissue specificity, which enhances its safety profile by creating a tissue-specific lesion while respecting the surrounding structures (e.g., coronary artery, esophagus, or phrenic nerve) [88]. However, to date, no in-human studies have yet to assess the effectiveness of electroporation in the context of VTs.

Recently, ultra-low-temperature cryoablation has been proposed to address the shortcomings in RF ablation, particularly in achieving transmurality of lesions in the left ventricle, which is often more than 6 mm thick [89]. This technique utilizes a nitrogen refrigerant at −196 °C [89]. A first-in-human study has shown promising results; however, the data are limited by the small number of enrolled patients, the extremely selective nature of the population enrolled (only monomorphic VTs), and the short follow-up duration (6 months) [89].

Finally, stereotactic ablation has shown interesting results. This approach is guided by non-invasive substrate mapping (CMR and MDCT) and consists in the high-precision delivering of radiation (photons) to the identified target [83]. However, safety concerns about the radiation effects have been raised, particularly regarding irradiation of the healthy tissue and organs adjacent to the target zone [90,91].

Thanks to its selective irradiation properties—extremely low entrance dose, absence of an exit dose, and a sharp fall in lateral doses—proton-beam ablation has shown promising results in preclinical studies. In swine heart models, a dose between 25 to 40 Gy was demonstrated both in imaging techniques (CMR) and in ex vivo analyses to be adequate for achieving scar homogenization at 30 weeks, while lower doses appear insufficient to guarantee an adequate effect [91,92,93].

## Figures and Tables

**Table 1:** Mapping strategies.

Mapping Approach	Procedure	Target	Strength	Weakness	Setting
Activation Mapping	Activation sequence during VT	Electrogram with the earliest activation (30–50 ms) Critical isthmus in reentrant	Directly identify arrhythmia source/circuit Not limited by functional line of block	70% of VTs are not tolerated	Enhanced automaticity and reentry
Pace Mapping	Ventricular pacing during sinus rhythm	Correspondence between paced QRS and clinical VT QRS	Identify either the exit site in focal arrhythmias or the mid-isthmus zone	Qualitative assessment Intramural circuits are not mappable Not specific for isthmus in reentrant VT Influenced by pacing threshold and rate	Enhanced automaticity and reentry
Entrainment Mapping	Entrain VT	Isthmus (PPI-TCL < 30 ms; S-QRS interval less than 50% TCL VT)	Identify isthmus	VT may not be tolerated Bystander sites may be confounding Antidromic activation of the circuit	Reentry
Substrate Mapping	Characterize arrhythmic substrate	LAVA Late potentials and isochrones Low-voltage zone	P2 distal to proximal during VT	Time consuming Not effective in functional blocks Far-field potentials and unstable contact lead to false results	Enhanced automaticity and reentry

VT: Ventricular tachycardia; PPI: Post-pacing interval; TCL: Tachycardia cycle length; LAVA: Local abnormal ventricular activity.

**Table 2:** Randomized clinical trials comparing the early vs. deferred ablation of ventricular tachycardia.

Study	Year	Author	Country	FU (Years)	N Patients	Group	LVEF	MI	Not MI	Death (All Causes)	VT Recurrence	Appropriate ICD Shock	BB	Amiodarone	Other AAD
PARTITA [65]	2012–2021	Della Bella et al.	Europe	2	47	Early ablation	31.9	20	3	0	7	2	23	1	-
						Control	32.4	18	6	8	12	10	24	4	-
BERLIN-VT [66]	2015–2018	Willems et al.	Europe Russia	1	159	Early ablation	41.6	83	-	6	29	13	58	31	-
						Control	41.6	72	-	2	40	18	59	22	-
VTACH [67]	2002–2006	Kuck et al.	Europe	2	107	Early ablation	31.9	20	3	0	7	2	23	1	-
						Control	34.1	55	-	4	39	26	41	19	-
SMASH-VT [68]	2000–2006	Reddy et al.	USA Czech Republic	2	128	Early ablation	31	64	-	6	8	6	60	0	-
						Control	33	64	-	11	21	20	63	0	-
SMS [69]	2002–2011	Kuck et al.	Europe	2	111	Early ablation	32	54	-	9	25	13	49	16	0
						Control	33	57	-	11	21	20	63	0	2
PAUSE-SCD [70]	2015–2020	Tung et al.	USA China	3	121	Early ablation	41	20	40	5	19	6	47	20	3
						Control	40	22	39	4	31	15	53	23	2

FU: Follow-up; LVEF: Left ventricle ejection fraction; MI: Previous myocardial infarction; VT: Ventricular tachycardia; ICD: Internal cardioverter–defibrillator; BB: Beta blockers; AAD: Antiarrhythmic drug.
